# Burden of respiratory syncytial virus infections in China: Systematic review and meta–analysis

**DOI:** 10.7189/jogh.05.020417

**Published:** 2015-12

**Authors:** Yaowen Zhang, Lichao Yuan, Yongming Zhang, Xiuping Zhang, Minghuan Zheng, Moe H Kyaw

**Affiliations:** 1 Infection Management and Disease Prevention Department, China–Japan Friendship Hospital, Beijing, China; 2 Department of Infectious Diseases, China–Japan Friendship Hospital, Beijing, China; 3 Department of Respiratory Diseases, China–Japan Friendship Hospital, Beijing, China; 4 China–Japan Friendship Clinical College, Beijing University of Chinese Medicine, Beijing, China; 5 Sanofi Pasteur, Beijing, China; 6 Sanofi Pasteur, Swiftwater, PA, USA

## Abstract

**Background:**

Respiratory syncytial virus (RSV) is the most important cause of acute respiratory tract infection (ARTI) related morbidity and mortality worldwide. However, the disease burden due to RSV has not been systematically summarized in China.

**Method:**

A systematic search was performed in the Chinese BioMedical Database (CBM), China National Knowledge Infrastructure (CNKI), Wanfang database and PubMed to identify available published RSV studies in China.

**Results:**

A total of 489 641 patients with ARTIs from 135 studies were included in the analysis. Among patients with ARTIs, RSV accounted for 18.7% (95% confidence interval CI 17.1–20.5%). The prevalence of RSV was highest in infants (26.5%, 95% CI 23.7–29.5%) and lowest in those aged ≥16 years (2.8%, 95% CI 1.3–6.1). A higher prevalence of RSV was seen in inpatients (22%, 95% CI 19.9–24.2%) than in outpatients (14%, 95% CI 9.6–19.9%). RSV type A accounted for 63.1% (95% CI 52.3–72.8%) of all RSV infections. RSV infections occurred mainly in winter and spring. The most common clinical manifestations were cough, production of sputum, wheezing and fever.

**Conclusion:**

RSV is the leading cause of viral ARTIs in China, particularly in infants and young children. Our findings are valuable for guiding the selection of appropriate therapies for ARTIs and implementation of preventive measures against RSV infections. Our data further supports the development of a successful RSV vaccine as a high priority.

Acute respiratory tract infections (ARTIs) are an important cause of morbidity and mortality among children under the age of 5 years [1,2], with the highest number of deaths occurring in developing countries [3]. In China, pneumonia is the leading cause of deaths in children under 5 years old with an estimated >30 000 deaths annually [4]. Viruses have been considered as the most frequent causes of ARTIs. The predominant viruses associated with ARTIs in children include respiratory syncytial virus (RSV), influenza virus (IV), parainfluenza virus (PIV), human rhinovirus (HRV) and adenovirus (ADV) [5,6].

RSV is the leading cause of ARTIs in early childhood. It is estimated that 33.8 million new episodes of RSV–associated ALRI occurred worldwide in children younger than 5 years, with at least 3.4 million episodes representing severe RSV–associated acute lower respiratory infection (ALRI) necessitating hospital admission [7]. The pattern of RSV infections is variable and related to season, socio–demographic and characteristics of study populations.

China has the largest child population and has substantial differences in climate from region to region. It has a variety of temperature and rainfall zones, including continental monsoon areas. The total population of children aged 14 years or younger is estimated to be 230 million. Although the epidemiology of RSV infections has been studied in cities such as Beijing, Chongqing and Lanzhou [8–10], few RSV studies in China have been published in English. Therefore, we performed a systematic review and meta–analysis of published studies to evaluate the epidemiology of RSV infections in patients with ARTIs.

A better understanding of the epidemiology of RSV infections plays a key role for the prevention, control and treatment of ARTIs. The objective of this systematic review and meta–analysis was to evaluate the etiology, serotypes, clinical features, age distribution and seasonality associated with RSV infections in China.

## METHODS

### Search strategy

A systematic search was performed in indexed databases, including Chinese BioMedical Database (CBM), China National Knowledge Infrastructure (CNKI), Wanfang database and PubMed to identify available RSV studies in China. The following search terms were used: RSV or syncytial virus. Taking into account the quality of studies, high quality publications from the Chinese core journals (2014 edition) [11] were considered in the final analysis. The Library of Perking University evaluates all Chinese journals every four years and excludes lower quality journals using the quality measurement similar to the impact factors. To obtain recent data, the search strategy was limited to publications dated from January 2010 to Mar 2015. Details of the search strategy are presented in Appendix S1 in **Online Supplementary Document(Online Supplementary Document)**.

### Inclusion and exclusion criteria

To be included, the following criteria had to be fulfilled: 1) studies in humans; 2) studies in patients with ARTIs; 3) studies that had at least one following outcome: etiology of acute respiratory infections; seasonality; gender; age group; serotypes; clinical features; 4) studies published in Chinese or English.

Publications were excluded if they were: 1) animal experiments or basic research (examples, studies focus on principles or mechanisms using cells and tissues); 2) case reports, systematic review or meta–analysis; 3) replicates (when the same population was studied in more than one publication, only the latest one or the one with the most complete data was considered for the meta–analysis).

ARTIs was defined as patients who were present of one or more respiratory symptoms, including watery eyes, rhinorrhea, nasal congestion or sinus congestion, otitis media, pharyngitis, cough, sore throat, sneezing, headache, and muscle pain. Meanwhile, patients had at least one symptom during acute infection, with high fever (body temperature ≥38°C) or chillness or normal/low leukocyte count or who were diagnosed with pneumonia by chest radiography previously. Chest radiography was conducted according to the clinical situation of the patient, and pneumonia was defined as an acute illness with radiographic pulmonary shadowing (at least segmental or in one lobe) by chest radiography.

### Literature screening and data extraction

Literature reviewers were divided into two parallel groups. Using the set criteria of inclusion and exclusion, the reviewers independently screened the literature by title, keyword and abstract. Any disagreement was solved by a third reviewer. If they were not sure whether the study should be included, the decision was made based on further review of the full texts. NoteExpress 2 (Aegean Software Corporation, Shanghai, China) was used for the bibliography management.

Two parallel groups independently extracted the following data from eligible studies: general information, methodological quality and outcome data. Inconsistencies between two groups were checked after data extraction. Any disagreements were solved by the third reviewer.

### Quality assessment

This meta–analysis included various types of studies with different outcomes. Therefore, no pre–existing scale is directly suitable for the assessment. The 5–item specific rating scale was developed to assess the quality of studies. These included 1) Did the study report patients’ information? 2) Did the study report diagnosis criteria of acute respiratory infection? 3) Did the study report specimen collection methods? 4) Did the study report pathogen detection methods? 5) Did the study report statistical methods? Each item was scored on three scales; 0 indicating low quality, 1 indicating medium quality, and 2 indicating high quality. The score for each item was then added to give a composite score for the study, with a highest total score of 10. If the total score was equal to or greater than 8, we regarded the study as “good” quality.

### Statistical analysis

The MetaAnalyst (Beta 3.13; Tufts Evidence–based Practice Center, Boston, USA) was used to conduct meta–analyses for pooled proportions and odds ratios. Considering heterogeneity across all studies, we chose a random–effects model to carry out meta–analysis using Der–Simonian Laird method. The publication bias was determined via Stata 12.0 (StataCorp LP, Texas, USA) using an Egger’s test. Meta–analysis for combining the results of studies was weighted to provide the balanced results of all included studies.

## RESULTS

### Selection of studies

**Figure 1:**
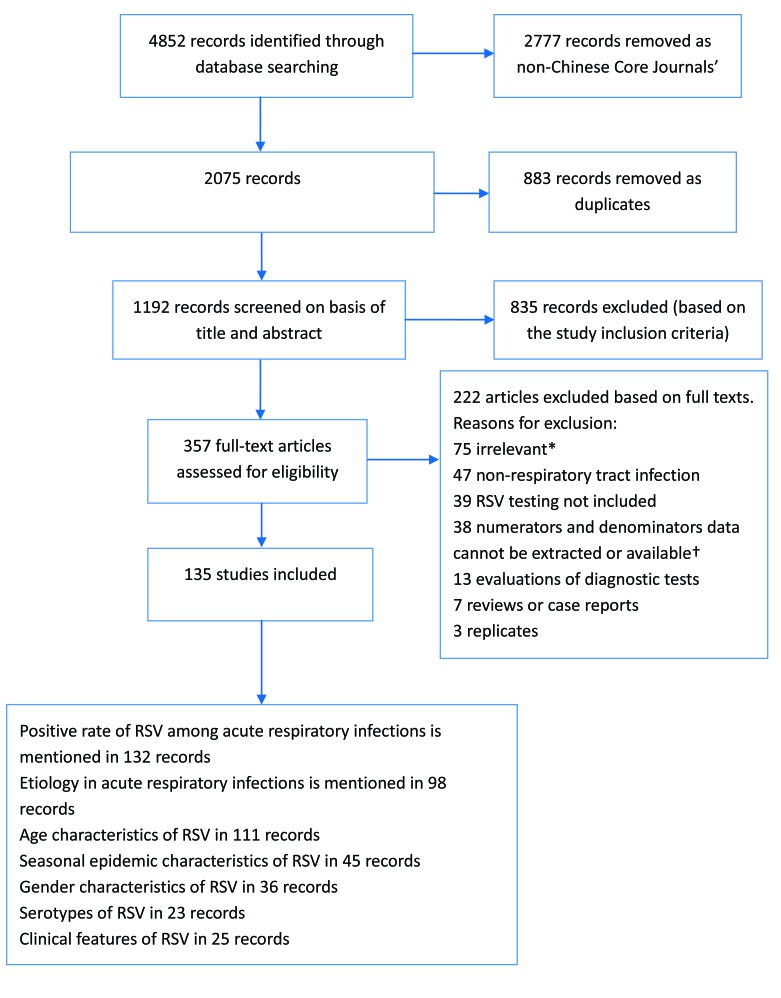
Flow diagram of literature search and selection. *Including: 1) Did not discuss respiratory tract infections or respiratory syncytial virus; or 2) only as background to discuss; or 3) used the abbreviation “RSV”, but not referring to respiratory syncytial virus. †These studies reported/included charts or risk ratios and the numerators and denominators were not available.

Of the total of 4852 studies identified through the databases, 135 studies were included in the analysis (**Figure 1**). Of the 135 studies, 123 studies were in children less than 16 years old and the remaining studies were in both children and adults. Of the 135 studies, 19 studies were published in English. The detailed information about author, publication year, province, age, specimen type, detection methodology, number of specimen and study outcomes are listed in Appendix S2 in **Online Supplementary Document(Online Supplementary Document)**.

For the 135 studies, the quality evaluation score ranged from 5 to 10 points, with a mean ± standard deviation of 7.7 ± 1.3. There were 80 studies with a score of ≥8. The summary of quality assessment is listed in Appendix S3 in **Online Supplementary Document(Online Supplementary Document)**.

### Etiology

The overall positivity rate of RSV among patients with ARTIs was 18.7% (95% CI 17.1–20.5%), followed by HRV, human bocavirus (HBoV), IV, PIV, human metapneumovirus (HMPV), enterovirus, ADV and human coronavirus (HCoV). (**Table 1**).

**Table 1:** Etiology of acute respiratory infection in all ages

Virus	No. articles included	Virus–positive	Total patients	Positive rate (%, 95% CI)
RSV	132	81 747	489 641	18.7 (17.1–20.5)
Rhinovirus	36	3647	31 605	11.5 (9.8–13.5)
HBoV	45	5899	110 345	6.8 (5.5–8.5)
IV	95	17 115	262 089	6.5 (5.4–7.7)
PIV	97	17 515	264 538	6.4 (5.6–7.2)
HMPV	59	5935	130 620	4.3 (3.6–5.1)
Enterovirus	16	923	17 689	4.0 (2.8–5.6)
Adenovirus	96	9618	275 380	3.4 (2.9–3.9)
HCoV	39	1544	66 048	2.6 (2.0–3.4)

RSV – respiratory syncytial virus, PIV – parainfluenza virus, IV – influenza virus, HMPV – human metapneumovirus, HBoV – human bocavirus; HCoV, Human coronavirus, CI – confidence interval

The prevalence of RSV was stratified into inpatients, outpatients, inpatients/outpatients and unknown category. RSV was found most frequently among inpatients 22.0% (95% CI 19.9–24.2%), followed by inpatients/outpatients, outpatients and unknown (**Table 2**).

**Table 2:** Positive rate of RSV infection in acute respiratory tract infected patients

	No. articles included	Virus–positive	Total patients	Positive rate (%, 95% CI)
**Settings:**				
Inpatient	86	67 319	366 386	22.0 (19.9–24.2)
Outpatient	9	1061	9229	14.0 (9.6–19.9)
Inpatient/outpatient	45	8779	67 826	15.8 (12.1–20.2)
Unspecified	95	4568	46 200	11.4 (7.4–17.1)
Total	132	81 747	489 641	18.7 (17.1–20.5)
**Age groups:**				
0–6m	45	12 522	43 222	29.9 (26.2–33.8)
0–1y	65	29 607	113 386	26.5 (23.7–29.5)
0–3y	55	27 544	130 152	23.7 (20.9–26.9)
0–6y	47	17 854	121 717	19.5 (16.0–19.6)
0–16y	72	57 193	351 426	17.7 (5.5–8.5)
≥16y	7	440	18 781	2.8 (1.3–6.1)
**Inpatients only by age groups:**				
0–6m	31	10 690	35 592	32.4 (27.7–37.4)
0–1y	44	25 259	97 542	34.4 (28.9–40.4)
0–3y	37	20 789	99 951	24.3 (20.6–28.3)
0–6y	28	12 446	88 248	19.8 (16.4–23.7)
0–16y	45	46 128	270 359	20.0 (17.9–22.3)
≥16y	3	174	8604	2.8 (0.8–9.8)
**Regions:**				
Northeast	2	285	3003	9.5 (8.5–10.6)
North China	18	2203	26 710	10.9 (6.3–18.4)
South China	23	10 625	96 413	15.7 (13.2–18.6)
East China	44	50 001	273 312	17.6 (15.3–20.2)
Central China	14	2978	16 346	22.5 (16.8–29.5)
Northwest	9	1523	5010	27.6 (21.3–34.9)
Southwest	20	10 375	35 970	28.7 (25.7–32.0)
Multi–regions	2	3757	32 877	14.7 (6.6–29.6)
Detection methodology*				
IF	51	41 201	230 033	17.0 (14.4–20.0)
PCR	73	32 814	201 063	19.9 (17.9–22.1)
Others	8	7732	58 545	20.1 (15.8–25.3)

*PCR – polymerase chain reaction, IF – immunofluorescence, Others – refers to PCR/IF and RVP Fast, y – year, m – month, CI – confidence interval

### Seasonal characteristics

A total of 45 studies reported the seasonality of RSV infections. Of these 45 studies, 28 studies reported monthly isolation rates and the remaining 17 studies reported quarterly. The peak of RSV infections mainly occurred in winter and spring (**Table 3**).

**Table 3:** Seasonal epidemic characteristics of RSV infection among acute respiratory infected patients

Month/season*	No. articles included	RSV–positive	Total patients	Positive rate (%) (95% confidence interval)
Jan	17	1150	4672	24.7 (15.2–37.5)
Feb	17	1094	4311	25.1 (17.1–35.4)
Mar	17	849	4003	19.5 (13.5–27.4)
Apr	16	504	3174	17.4 (13.5–22.3)
May	15	181	2551	8.7 (6.1–12.3)
Jun	14	135	2945	5.5 (3.1–9.4)
Jul	14	105	2805	4.5 (3.–6.8)
Aug	13	163	2605	7.0 (4.5–10.7)
Sep	14	183	2511	6.4 (3.6–11.3)
Oct	16	254	2935	7.5 (4.2–13.1)
Nov	17	597	3522	14.4 (8.8–22.5)
Dec	17	1049	3954	26.2 (17.9–36.8)
Spring	27	5627	37 738	13.9 (11.1–17.4)
Summer	27	2008	31 716	5.3 (3.7–7.6)
Autumn	27	4204	28 223	11.8 (8.2–16.8)
Winter	27	9774	32 937	22.7 (17.4–29.1)

*Spring – 1 March to 31 May, Summer – 1 June to 31 August, Autumn – 1 September to 30 November, Winter – 1 December to 28/29 February.

### Gender characteristics

A total of 36 studies reported gender characteristics of RSV infections. Among 96 694 male patients, RSV was positive in 17 163 patients (20.4%, 95% CI 16.6–24.8%). Among 54 958 female patients, RSV was positive in 8364 (19.9%, 95% CI 16.0–24.4%).

### RSV serotypes

There were 23 studies that reported RSV serotypes. Among 4172 RSV infected patients; type A, type B and mixed type A and B were detected in 2469 (63.1%, 95% CI 52.3–72.8%), 1611 (35.6%, 95% CI –6.0 to –46.6%) and 92 (1.2%, 95% CI 0.7–2.2%), respectively.

### Clinical characteristics

Twenty–five studies reported the clinical manifestations of RSV infection. The most frequently reported clinical manifestations were cough, sputum production, wheezing and fever (**Table 4**).

**Table 4:** Clinical characteristics of RSV related acute respiratory infected patients

Clinical characteristics	No. articles included	Symptomatic patients	RSV infected patients	Percent (95% CI)
Cough	24	9880	11 194	93.9 (91.0–96.0)
Expectoration	6	1578	2316	66.3 (43.8–83.2)
Wheeze	13	1768	2778	65.7 (56.5–73.8)
Fever	22	3738	10 018	43.0 (37.5–48.7)
Rhinorrhea	8	1621	5669	42.7 (31.6–54.6)
Cyanosis	9	1346	4318	38.9 (15.8–68.2)
Tachypnea	11	2024	7737	32.2 (15.7–54.9)
Diarrhea	7	838	5272	18.8 (11.4–29.5)
Dyspnoea	8	1707	7918	12.8 (5.0–28.9)

### Sensitivity analysis and publication bias

The conclusions remained robust and the outcomes did not alter significantly when only ‘good’ quality studies were evaluated in the sensitivity analysis. The overall RSV positivity rates were 19.7% (95% CI 17.7–21.9%) in all patients (n = 79), 22.7% (95% CI 20.2–25.3%) in inpatients (n = 53), and 13.8% (95% CI 8.3–22.0%) in outpatients (n = 5).

Publication bias was tested using the Egger’s test. No publication bias was detected when verifying the 132 publications that reported RSV positive rates in all patients (–0.27, 95% CI –1.19 to –0.65, *P* = 0.563).

## DISCUSSION

RSV is the most common viral cause of ARTIs in developed and developing countries [7,12]. However, the available epidemiological data on RSV in China has not been systematically summarized in English. Our results highlight that RSV is the leading cause of viral ARTIs in China.

The burden of respiratory viral infections is difficult to measure and is likely to differ from country to country due to several factors such as socio–demographic distribution, seasonal variation, study design and diagnostic techniques. In our study, RSV was the most frequently detected pathogen among patients with ARTIs in all age groups studied. Consistent with other studies, RSV and HRV were the most prevalent viruses in children [2,13,14].

In the present study, only 12 studies reported the RSV infections in adult patients and the remaining 123 studies were in children. Infants aged ≤1 year were at higher risk of RSV associated ARTIs, compared with those in other age groups. This is consistent with the previous reports of RSV in both developed and developing countries [7,12,15,16]. The data show that RSV accounted for nearly 30% of all ARTIs in infants. Efforts to prevent RSV infections in infants can lead to a substantial reduction of RSV associated morbidity, mortality and medical costs in China. Further evidence of RSV disease burden can be established by adding RSV studies in existing influenza surveillance systems.

The relation between RSV infections and climate has been well documented [17,18]. In regions with persistently warm temperatures and high humidity, RSV activity is continuous throughout the year, peaking in summer and early autumn. In temperate climates, RSV activity is maximal during winter, correlating with lower temperatures. In areas where temperatures remain colder throughout the year, RSV activity also occurs almost continuously [17,18]. Most areas of China have a temperate climate. We found that the peak of RSV activity mainly occurred during winter and spring in China, which is similar to the previous reports [19–21]. This pattern of seasonality corresponds to the cold and dry seasons. However, we are unable to report regional differences in RSV activity due to limited data availability.

Based on genetic and antigenic variations in structural proteins, RSV isolates are subdivided into two major antigenic types (A and B). Both types are associated with mild to severe ARTIs [22–24]. Studies have shown that type A and B viruses co–circulate in the same area during epidemic periods and have various patterns of predominance [25–28]. However, the prevalence of each type may shift yearly and can vary among different communities [28–30]. Our analysis showed that type A was the predominant serotype accounting for 63.1% of all RSV infections. However, it is difficult to know how the sero–epidemiological trend changed in the recent years in China. This is because the study periods and locations varied substantially among included studies.

The studies we included differed in their methods of sampling and case–definition. Therefore, caution should be taken when interpreting the results. In our study, only 42 studies presented detailed criteria for case–definition. In addition, only 54 out of the 135 included studies used immunofluorescence for RSV detection. RSV was identified less frequently (17.0%) if only the results of studies based on immunofluorescent detection were included. In comparison with immunofluorescence, molecular diagnostics are more sensitive and specific [31,32]. In recent years, the introduction of nucleic acid based diagnostic tests has markedly improved our understanding of viral etiology among ARTI patients [33]. Therefore, the real burden of RSV in China is likely to be higher than our findings if more sensitive diagnostic methods are used.

These differences in case definitions and diagnostic techniques are likely to have impacted the results. Therefore, a random effects model was applied to take into account the heterogeneity between studies resulting in wider 95% CIs with more conservative estimates of the overall results [34]. In addition, the etiology data in the present study should be interpreted with caution. This is because restricting RSV to the title and abstract in the search criteria is a potential source of bias and might not be representative of all studies reporting other viruses. However, all included studies in current review tested for multiple viruses. Therefore, it is reasonable to assume that the use of correct denominator and numerators allow us to present the useful and informative etiology data available in these studies.

In conclusion, this systematic review and meta–analysis showed that RSV is the leading cause of ARTIs in China, particularly among infants. Our findings are valuable for guiding the selection of appropriate therapies for ARTIs and implementation of preventive measures against RSV infections. Despite the disease burden, no effective RSV vaccine is currently available. Our data further supports the development of a successful RSV vaccine as a high priority.
