# Supplementary material for: Burden of respiratory syncytial virus infections in China: Systematic review and meta–analysis
Source: J Glob Health. 2015 Dec 2;5(2):020417. doi: 10.7189/jogh.05.020417 (PMC4676581; doi:10.7189/jogh.05.020417)
Supplement: Online Supplementary Document [file jogh-05-020417-s001.pdf]

Online Supplementary Document

Zhang et al. Burden of respiratory syncytial virus infections in China: Systematic review and meta–analysis

J Glob Health 2015;5:010417

Appendix 1. Search strategy and results

Total literatures : 4852   21 Mar, 2015

1）CHKD：1607 篇

SU=("合胞病毒") or SU=("合胞体病毒") or SU=("RSV") or SU=("Syncytial Virus")

学科领域：医药卫生科技
检索日期：2010/01/01-2015/03/21

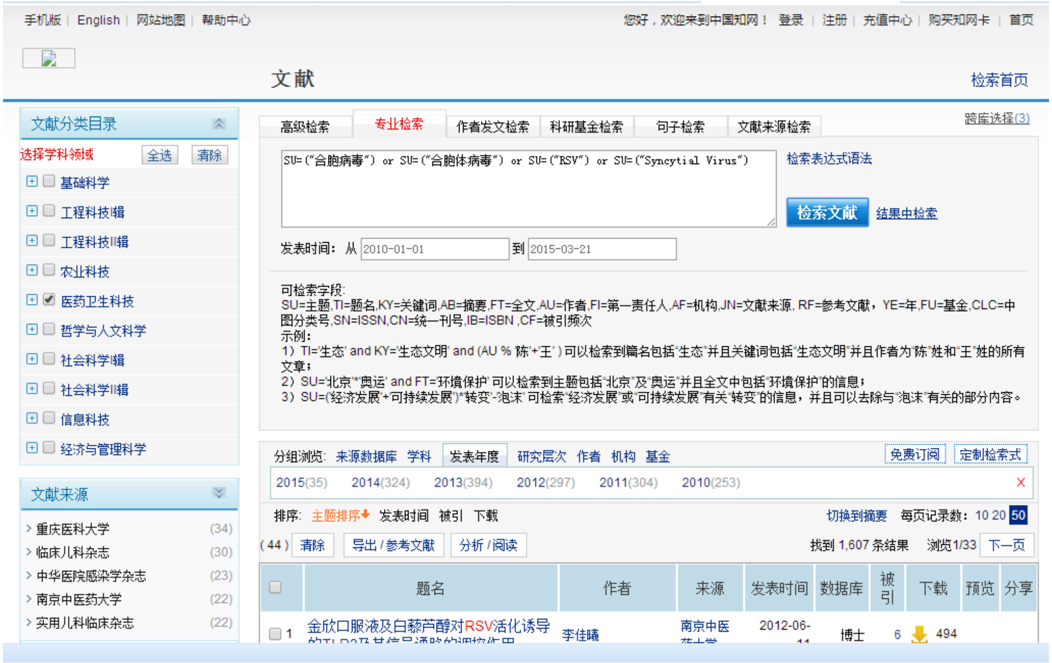

2）万方：1514 篇

主题:("合胞病毒")+主题:("合胞体病毒")+主题:("RSV")+主题:("Syncytial Virus")
分类号:"R*"

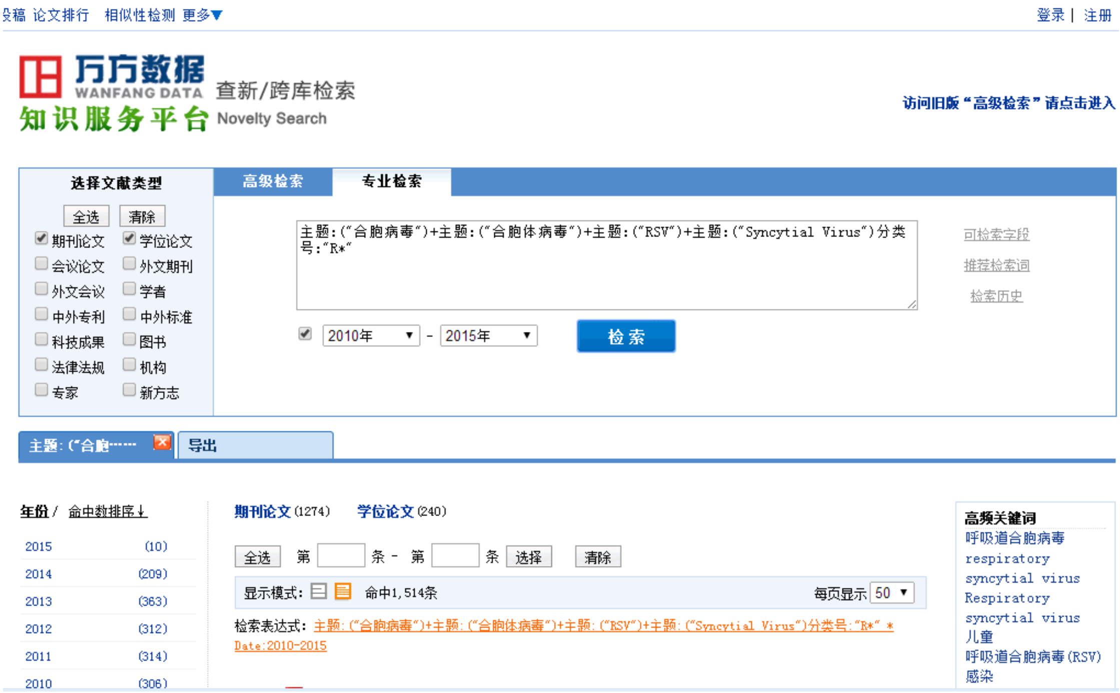

3）中国生物医学文献（CBM）：1643 篇

OR "合胞病毒"[常用字段:智能]) OR "合胞体病毒"[常用字段:智能]) OR "RSV"[常用字段:智能]) OR "Syncytial Virus"[常用字段:智能]

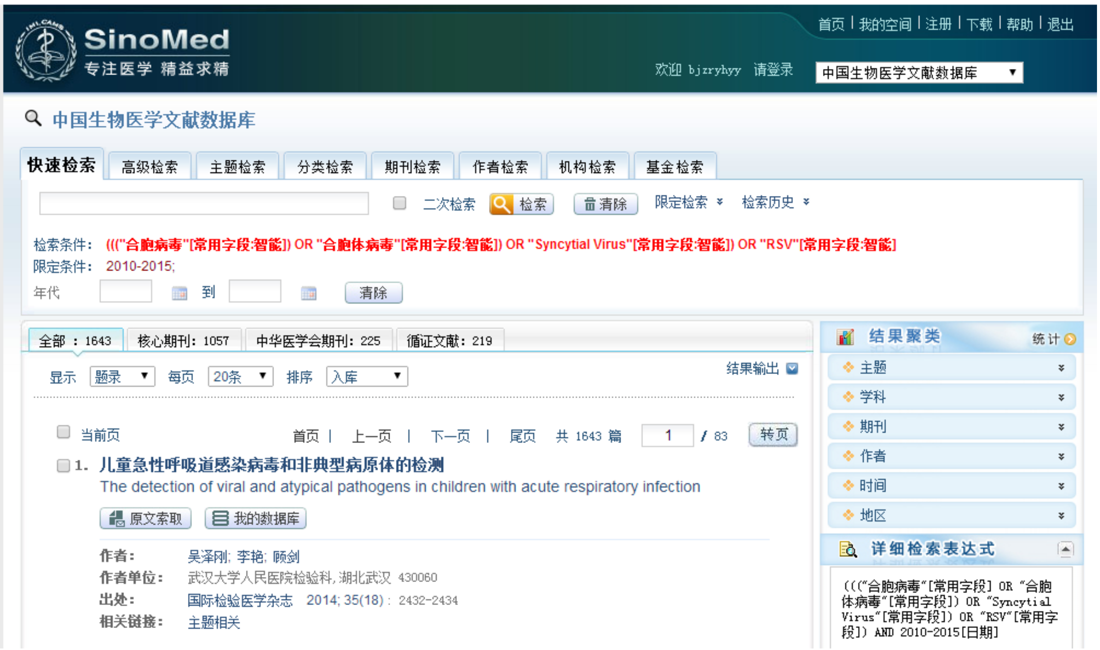

4）PubMed：88 篇

(((Syncytial virus[Title/Abstract]) OR RSV[Title/Abstract])) AND

(((Chinese[Title/Abstract]) OR China[Title/Abstract]) OR China[MeSH Terms]) Filters: published in the last 5 years; Humans

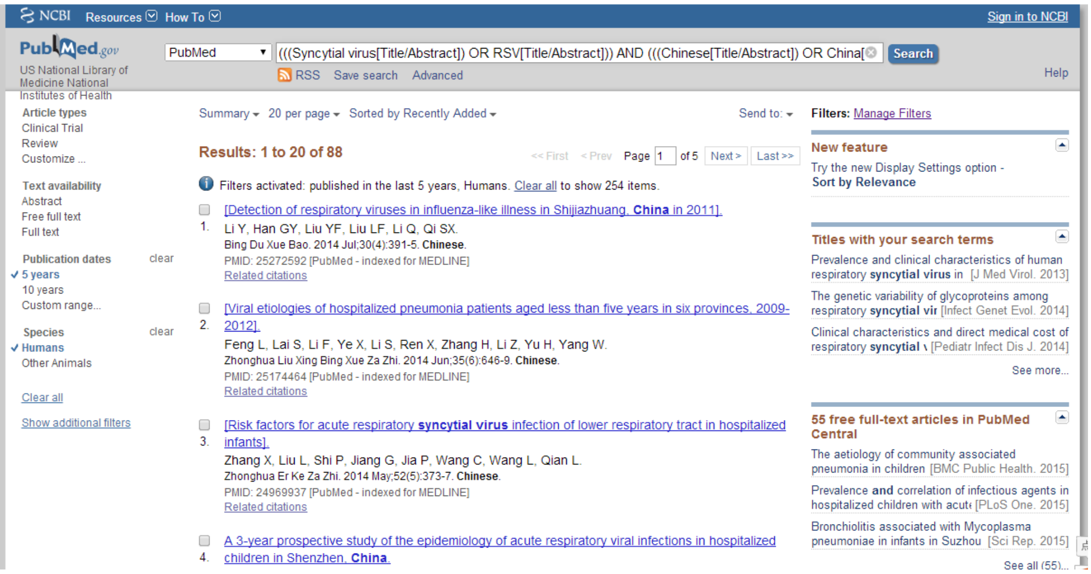

Appendix 2. Summary of articles used for meta-analysis

Author	Year	Province	Patient source	Age	Specimen type*	Detection methodology†	No. of Specimen	Outcomes‡
Su YQ	2010	Fujian	Outpatient/Inpatient	0-96y	NPAs	PCR	153	1,6
Du LN	2010	Chongqing	Inpatient	<12y	NPAs	PCR	508	1,2,4,5
Zhang GC	2011	Shanghai	Outpatient	<1y	NPAs	PCR	62	1,2
Ji J	2014	Jiangsu	Inpatient	<15y	NPAs	PCR	42208	1,2,5
Cheng Y	2014	Guangdong	Inpatient	<5y	NPAs	PCR	627	1,2
Wan FG	2013	Jiangsu	Inpatient	<15y	NPAs	IF	28871	1,2
Liang Y	2012	Zhejiang	Inpatient	0-1m	NPAs	IF	75	6
Fan L	2013	Guizhou	Inpatient	0-1y	NPAs	IF	551	1,2,4,6
Cai BB	2013	Zhejiang	Inpatient	0-3y	Sputum	IF	82	6
Zhi M	2011	Xinjiang	Inpatient	Children	NPAs	PCR	392	1,2,4,5
Feng ZL	2014	Multi-Center	Inpatient	0-5y	NPAs/Sputum	PCR	4508	1,2,4,6
Yu CM	2010	Chongqing	Inpatient	0-4y	NPAs	PCR	119	1,2
Zhu Y	2011	Chongqing	Inpatient	0-1m	NPAs	IF	709	1,2,4,6
Zhang DM	2013	Guangdong	Outpatient/Inpatient	0-110y	NPAs	PCR	14227	1,2,4
Tian M	2010	Jiangsu	Inpatient	0-12y	NPAs	IF	5480	1,2,3
Ji W	2011	Jiangsu	Inpatient	0-10y	NPAs	PCR and IF	6655	1,2,3
Cao Y	2013	Sichuan	Inpatient	0-13y	NPAs	PCR	592	1,2,3,4,5
Kong M	2011	Tianjin	Inpatient	0-12y	NPAs	PCR	202	1,2,3
Wang W	2012	Tianjin	Inpatient	0-12y	NPAs	IF	5954	1,2,3,5
Wu H	2012	Xizang	Inpatient	0-13y	NPAs	IF	167	1,2,3
Ning J	2011	Tianjin	Outpatient/Inpatient	0-1y	NPAs	PCR and IF	187	1,2
Lu XM	2012	Guangdong	Inpatient	0-12y	NPAs	IF	1256	1,2,3
Zou LR	2011	Guangdong	Outpatient/Inpatient	Children and Adults	NPAs	PCR	1554	1
Jiang YW	2013	Jiangsu	Inpatient	0-5y	NPAs	PCR and IF	474	1,2,4

Chen Y	2011	Guangdong	Outpatient/Inpatient	0-14y	NPAs	PCR	21458	1,2
Wang YQ	2011	Jiangsu	Inpatient	0-10y	NPAs	PCR and IF	6655	1,2
Zhou ZG	2011	Guangdong	Outpatient	0-88y	NPAs	PCR	1388	1
Chen XW	2011	Guangdong	Outpatient/Inpatient	0-14y	NPAs	PCR	763	1,2,3,4
Ding XF	2012	Hunan	Inpatient	0-6y	NPAs	PCR	100	1,2,4
Hua W	2010	Zhejiang	Inpatient	0-1y	NPAs	IF	8511	1,2
Zhao YF	2013	Jiangsu	Inpatient	0-14y	NPAs	IF	7830	1
Huang DJ	2012	Heilongjiang	Inpatient	17-90y	NPAs	IF	521	1,2,3
Chen Q	2010	Jiangsu	Inpatient	0-14y	NPAs	IF	1592	1,2,3
Zhou ZG	2010	Guangdong	Outpatient	0-14y	NPAs	PCR	763	1,2,3
Xie HM	2012	Shanghai	Outpatient/Inpatient	13-98y	Blood	IF	1647	1,2,3
Xiao NG	2012	Hunan	Inpatient	0-14y	NPAs	PCR	1165	1,2,4
Chang J	2010	Zhejiang	Inpatient	0-13y	NPAs	IF	5097	1,2,3
Ding XF	2012	Hunan	Inpatient	0-6y	NPAs	PCR	100	1,2
Zheng H	2015	Zhejiang	Outpatient/Inpatient	Children	Blood	IF	2119	1,2,3
Wu ZG	2011	Hubei	not specified	0-15y	Blood	IF	947	1,2
Yang XH	2013	Guangdong	Outpatient/Inpatient	0-14y	NPAs	IF	9459	1,2,3
Ding GB	2013	Jiangxi	Inpatient	0-6y	Blood	IF	918	1,2
Liu YF	2013	Hebei	Outpatient	0-5y	NPAs	PCR	178	1,2,4,5
Guo L	2014	Xinjiang	Outpatient/Inpatient	Children	NPAs	PCR	500	1,2
Zhu MH	2013	Guangdong	Outpatient/Inpatient	Children	NPAs	PCR	720	1,2
Xue B	2014	Jiangsu	not specified	Children and Adults	Blood	IF	1568	1
Jin YX	2013	Zhejiang	Inpatient	0-13y	NPAs	IF	8594	1,2
Huang L	2013	Guizhou	Inpatient	0-14y	NPAs	IF	1396	1,2,3,4
Fu JJ	2013	Jiangxi	Inpatient	0-14y	NPAs	IF	2276	1,2,3
Yan HJ	2011	Shanghai	Outpatient	0-6y	NPAs	PCR	843	1,2

Ding GB	2011	Jiangxi	Inpatient	0-6y	Blood	IF	151	1,2,3
Ai HW	2012	Hubei	not specified	0-16y	Blood	IF	6348	1,2,3
Xu LL	2014	Anjui	not specified	0-15y	not specified	IF	9693	1,3,4
Hu FR	2010	Zhejiang	Inpatient	0-6y	NPAs	IF	2221	1,2
Wang C	2012	Zhejiang	Inpatient	0-14y	Blood	IF	1375	1,2,3
Jin SM	2011	Zhejiang	Outpatient/Inpatient	0-5y	NPAs	IF	431	1,2
Ru GP	2013	Shanxi	Inpatient	0-5y	NPAs	IF	1200	1,2
Yin F	2014	Jiangsu	Inpatient	0-15y	NPAs	IF	15328	1,2,3
Zhu Y	2011	Chongqing	Inpatient	0-1m	Sputum	IF	709	1,2,3,4,6
Li BQ	2012	Jiangsu	Inpatient	0-14y	NPAs	PCR and IF	5632	1,2,3,6
Mao XJ	2010	Guangdong	Inpatient	0-14y	Blood	IF	12195	1,2,3,4
Lin J	2011	Zhejiang	Inpatient	0-5y	NPAs	IF	20986	1,2,6
Lei XY	2012	Chongqing	Inpatient	0-17y	NPAs	IF	19452	1,2,3,4
Ye YN	2011	Guangdong	Inpatient	Children	NPAs	PCR	1701	1
Du LN	2010	Chongqing	Inpatient	0-12y	NPAs	PCR	508	1,2,5
Feng JH	2012	Guangdong	Inpatient	Children	NPAs	PCR	1335	1
Xie SX	2010	Guangdong	Inpatient	Children	NPAs	PCR	271	1,4
Lu QB	2013	Chongqing	Inpatient	0-16y	NPAs	PCR	2451	1,2,3,4,5,6
Peng Y	2014	Hunan	Inpatient	0-13y	NPAs	PCR	653	1,2,3,4
Zhang HJ	2014	Liaoning	Inpatient	0-4y	NPAs	IF	2482	1,2,3,4
Zhao Y	2010	Gansu	Outpatient/Inpatient	0-14y	NPAs	PCR	510	1,2
Peng CJ	2012	Chongqing	Inpatient	0-16y	NPAs	PCR	1745	1,2
Liu Y	2013	Hubei	Inpatient	0-7y	NPAs	PCR	544	1,2,3,4
Qin X	2012	Chongqing	Inpatient	Children	NPAs	PCR	921	1,5,6
Xiao NG	2011	Hunan	Inpatient	0-14y	NPAs	PCR	1165	1,2,3,4
He LY	2013	Chongqing	Inpatient	0-1m	NPAs	IF	286	6

Deng J	2012	Xizang	Inpatient	Children	NPAs	PCR	167	1,5
Wang MJ	2013	Jiangsu	Inpatient	Children	NPAs	IF	1885	1,6
Zhou LL	2014	Chongqing	Inpatient	0-2y	NPAs	PCR	454	1,2,5
Zhang XB	2013	Shanghai	Inpatient	0-1y	NPAs	IF	1726	1,2,3,4
Lu AT	2014	Neimenggu	not specified	0-14y	NPAs	PCR	3207	1,2,3,4
Cao HY	2013	Gansu	Inpatient	0-14y	NPAs	PCR	545	1,4,6
Zheng WJ	2011	Yunnan	Inpatient	0-5y	NPAs	PCR	388	1,2,4,5,6
Wang SL	2012	Guangdong	Inpatient	1-14y	NPAs	PCR	593	1,2,6
Lu Q	2012	Beijing	not specified	0-3y	NPAs	PCR	329	1,2,5
Yan HJ	2014	Shanghai	Outpatient	0-12y	NPAs	PCR	4389	1,2
Yang Z	2015	Jiangsu	not specified	0-1m	NPAs	IF	872	1,2,4,6
Feng LZ	2014	Multi-Center	Inpatient	Children and Adults	NPAs/Sputum	PCR and IF	28369	1,2,6
Xue YM	2012	Gansu	Inpatient	0-14y	Blood	IF	768	1,2,4,6
Zhao X	2012	Hunan	Inpatient	0-14y	NPAs	PCR	771	1,2,3,4
Xiao X	2014	Guangdong	Outpatient/Inpatient	0-14y	Blood	IF	1447	1,2,4,6
Chen XQ	2010	Jiangsu	Inpatient	0-14y	NPAs	PCR	340	1,2
Zhi M	2011	Xinjiang	Inpatient	0-14y	NPAs	IF	309	1
Liu CY	2012	Beijing	Outpatient	0-15y	NPAs	PCR	540	1,2,3,5
Jiang M	2013	Jiangsu	Outpatient/Inpatient	0-14y	NPAs	IF	7459	1,2
Zou M	2013	Tianjin	Outpatient	0-14y	NPAs	PCR	249	1,2,3,5
Wang YQ	2012	Jiangsu	Inpatient	0-16y	Sputum	PCR	6450	1,2,3
Zhang HQ	2015	Guangxi	Inpatient	0-11y	NPAs	IF	3496	1,2,3
Mao XJ	2010	Guangdong	Inpatient	0-14y	Blood	IF	12195	1,2,3,4
Shi WX	2012	Beijing	Outpatient/Inpatient	Children and Adults	NPAs/Sputum	PCR	501	1,2,3
Ding JL	2013	Zhejiang	Inpatient	0-12y	NPAs	IF	2129	1,2
Liu H	2014	Hunan	Inpatient	0-14y	NPAs	PCR	697	1,2

He Y	2015	Sichuan	Outpatient/Inpatient	Children	NPAs	PCR	1025	1,2
Li L	2013	Jiangsu	Inpatient	0-14y	Blood	IF	2543	1,2,3
Chen HX	2014	Zhejiang	not specified	0-14y	Blood	IF	2427	1,2,3
Jiang YW	2013	Jiangsu	Inpatient	0-5y	NPAs	PCR	1126	1,2
Zhu R	2014	Beijing	Outpatient/Inpatient	0-6y	NPAs	PCR	270	1,2
Zhang XB	2014	Shanghai	Inpatient	0-1y	NPAs	IF	1726	1,2,3,4
Zhang QL	2014	Guangdong	Inpatient	0-12y	NPAs	IF	1383	1,2
Wang XH	2014	Beijing	Outpatient/Inpatient	Children	NPAs/Sputum	PCR	93	1
Ji W	2013	Jiangsu	Inpatient	0-14y	NPAs	PCR and IF	10243	1,2,3
Liu CY	2013	Beijing	Outpatient/Inpatient	Children	NPAs	PCR	2066	1,2
Feng JH	2012	Guangdong	Inpatient	Children	NPAs	PCR	1335	1
Zhang L	2012	Beijing	Inpatient	50y-93y	Blood	IF	351	1,2
Wang W	2012	Tianjin	Inpatient	0-1m	NPAs	IF	766	1,2,3
Qian XB	2010	Zhejiang	Outpatient/Inpatient	0-7y	NPAs	IF	517	1,2
He Y	2014	Guangdong	Inpatient	0-14y	NPAs	PCR	2025	1,2
Liu WK	2014	Guangdong	not specified	0-14y	NPAs	PCR	4242	1,2
Cai XY	2014	Guangdong	Inpatient	0-12y	NPAs	PCR	1980	1,2
Lu Y	2013	Shandong	Outpatient/Inpatient	0-14y	NPAs	PCR	720	1,2,5
Huo X	2013	Hubei	not specified	0-5y	NPAs	PCR	511	1,2,4
Xia Q	2014	Chongqing	not specified	0-12y	NPAs	PCR	1800	1,2,5
Zhou W	2013	Zhejiang	Inpatient	0-11y	Sputum	PCR	273	1,2
Zhang Q	2013	Jiangsu	Inpatient	0-14y	NPAs	PCR	295	1,2
Zhang XL	2013	Jiangsu	Inpatient	0-15y	NPAs	PCR	42664	1,2,4
Zhang C	2013	Beijing	Inpatient	0-8y	NPAs	RVP Fast	330	1,2,5
Huang GH	2013	Gansu	not specified	0-12y	NPAs	PCR	279	1,2,5
Lu Y	2013	Shandong	not specified	14-86y	NPAs	PCR	596	1,2

Qin X	2013	Chongqing	Inpatient	Children	NPAs	PCR	921	1,5,6
Xiang Z	2013	Beijing	not specified	≥15y	NPAs	PCR	9871	1,4,6
Yu X	2012	Beijing	not specified	＞14y	NPAs	PCR	416	1
Jin Y	2012	Gansu	not specified	0-14y	NPAs	PCR	813	1,2
Wang W	2010	Shanghai	Outpatient	0-9y	NPAs	PCR	817	1,2,5,6
Zhang RF	2010	Gansu	not specified	0-14y	NPAs	PCR	894	1,5,6
Zhang ZY	2010	Chongqing	not specified	Children	NPAs	PCR	1387	1,5,6

* NPAs, nasopharyngeal aspirates.

† PCR, polymerase chain reaction; IF, immunofluorescence.

‡ 1=Etiology; 2=Age characteristics; 3=Seasonal epidemic characteristics; 4=Gender characteristics; 5=Serotypes; 6=Clinical characteristics.

Appendix 3. Summary of quality assessment for all included studies

Author	Year	Province	Q1*	Q2	Q3	Q4	Q5	Total
Su YQ	2010	Fujian	2	1	2	2	1	8
Du LN	2010	Chongqing	2	0	1	2	0	5
Zhang GC	2011	Shanghai	2	0	1	2	2	7
Ji J	2014	Jiangsu	1	0	2	2	1	6
Cheng Y	2014	Guangdong	2	1	2	1	2	8
Wan FG	2013	Jiangsu	1	0	1	2	2	6
Liang Y	2012	Zhejiang	2	2	2	2	1	9
Fan L	2013	Guizhou	2	0	1	1	1	5
Cai BB	2013	Zhejiang	1	2	1	1	1	6
Zhi M	2011	Xinjiang	2	0	1	2	1	6
Feng ZL	2014	Multi-Center	2	2	2	2	2	10
Yu CM	2010	Chongqing	2	2	2	2	1	9
Zhu Y	2011	Chongqing	2	0	1	1	1	5
Zhang DM	2013	Guangdong	2	2	1	2	1	8
Tian M	2010	Jiangsu	2	1	2	2	1	8
Ji W	2011	Jiangsu	2	1	2	2	2	9
Cao Y	2013	Sichuan	2	0	1	2	1	6
Kong M	2011	Tianjin	2	0	1	2	1	6
Wang W	2012	Tianjin	2	0	2	1	1	6
Wu H	2012	Xizang	2	1	2	2	1	8
Ning J	2011	Tianjin	2	1	2	2	1	8
Lu XM	2012	Guangdong	2	1	2	2	1	8
Zou LR	2011	Guangdong	2	2	2	2	2	10
Jiang YW	2013	Jiangsu	2	2	2	2	1	9
Chen Y	2011	Guangdong	2	0	1	2	0	5
Wang YQ	2011	Jiangsu	2	0	2	2	2	8
Zhou ZG	2011	Guangdong	2	1	2	2	1	8
Chen XW	2011	Guangdong	2	1	2	2	1	8
Ding XF	2012	Hunan	2	2	2	2	1	9
Hua W	2010	Zhejiang	1	1	2	2	1	7
Zhao YF	2013	Jiangsu	2	0	2	2	1	7
Huang DJ	2012	Heilongjiang	2	0	2	2	1	7
Chen Q	2010	Jiangsu	2	1	2	2	1	8
Zhou ZG	2010	Guangdong	2	2	2	2	1	9
Xie HM	2012	Shanghai	2	0	1	2	1	6
Xiao NG	2012	Hunan	2	1	2	2	1	8
Chang J	2010	Zhejiang	2	1	1	2	1	7
Ding XF	2012	Hunan	2	2	2	2	2	10
Zheng H	2015	Zhejiang	2	0	2	2	1	7
Wu ZG	2011	Hubei	1	0	2	2	1	6
Yang XH	2013	Guangdong	2	0	2	2	1	7

Ding GB	2013	Jiangxi	2	1	2	2	1	8
Liu YF	2013	Hebei	2	0	2	2	1	7
Guo L	2014	Xinjiang	2	0	2	2	1	7
Zhu MH	2013	Guangdong	2	1	2	2	2	9
Xue B	2014	Jiangsu	1	0	1	2	1	5
Jin YX	2013	Zhejiang	2	0	2	2	1	7
Huang L	2013	Guizhou	2	0	2	2	1	7
Fu JJ	2013	Jiangxi	2	1	2	2	2	9
Yan HJ	2011	Shanghai	2	1	1	2	1	7
Ding GB	2011	Jiangxi	2	1	1	2	1	7
Ai HW	2012	Hubei	1	1	2	2	2	8
Xu LL	2014	Anjui	1	0	0	2	2	5
Hu FR	2010	Zhejiang	2	0	2	2	0	6
Wang C	2012	Zhejiang	2	2	2	2	1	9
Jin SM	2011	Zhejiang	2	0	2	2	1	7
Ru GP	2013	Shanxi	2	1	2	2	1	8
Yin F	2014	Jiangsu	2	0	2	2	2	8
Zhu Y	2011	Chongqing	2	2	2	2	1	9
Li BQ	2012	Jiangsu	2	1	2	2	1	8
Mao XJ	2010	Guangdong	2	2	1	2	2	9
Lin J	2011	Zhejiang	2	2	2	2	2	10
Lei XY	2012	Chongqing	2	0	2	2	1	7
Ye YN	2011	Guangdong	2	1	2	2	2	9
Du LN	2010	Chongqing	2	0	2	2	2	8
Feng JH	2012	Guangdong	2	2	2	2	2	10
Xie SX	2010	Guangdong	2	1	2	2	2	9
Lu QB	2013	Chongqing	2	2	2	2	2	10
Peng Y	2014	Hunan	2	2	2	2	1	9
Zhang HJ	2014	Liaoning	2	1	2	2	1	8
Zhao Y	2010	Gansu	2	0	1	1	1	5
Peng CJ	2012	Chongqing	2	2	2	2	2	10
Liu Y	2013	Hubei	2	2	2	2	2	10
Qin X	2012	Chongqing	2	0	2	2	2	8
Xiao NG	2011	Hunan	2	1	2	2	1	8
He LY	2013	Chongqing	2	2	1	1	1	7
Deng J	2012	Xizang	2	0	0	2	1	5
Wang MJ	2013	Jiangsu	2	0	2	2	1	7
Zhou LL	2014	Chongqing	2	0	2	2	1	7
Zhang XB	2013	Shanghai	2	2	2	2	2	10
Lu AT	2014	Neimenggu	1	2	2	2	2	9
Cao HY	2013	Gansu	2	1	2	2	1	8
Zheng WJ	2011	Yunnan	2	2	2	2	1	9
Wang SL	2012	Guangdong	2	2	2	2	2	10
Lu Q	2012	Beijing	1	1	2	2	1	7

Yan HJ	2014	Shanghai	2	2	2	2	1	9
Yang Z	2015	Jiangsu	1	2	1	1	1	6
Feng LZ	2014	Multi-Center	2	2	2	2		8
Xue YM	2012	Gansu	2	1	2	2	1	8
Zhao X	2012	Hunan	2	1	2	2	1	8
Xiao X	2014	Guangdong	2	0	1	2	1	6
Chen XQ	2010	Jiangsu	2	1	2	2	0	7
Zhi M	2011	Xinjiang	2	0	2	2	1	7
Liu CY	2012	Beijing	2	0	2	2	0	6
Jiang M	2013	Jiangsu	2	0	2	2	1	7
Zou M	2013	Tianjin	2	1	1	2	1	7
Wang YQ	2012	Jiangsu	2	0	2	2	2	8
Zhang HQ	2015	Guangxi	2	1	2	2	1	8
Mao XJ	2010	Guangdong	2	1	1	1	1	6
Shi WX	2012	Beijing	2	1	1	2	1	7
Ding JL	2013	Zhejiang	2	0	1	2	1	6
Liu H	2014	Hunan	2	2	2	2	1	9
He Y	2015	Sichuan	2	1	2	2	2	9
Li L	2013	Jiangsu	2	0	1	2	1	6
Chen HX	2014	Zhejiang	1	0	1	2	1	5
Jiang YW	2013	Jiangsu	2	1	2	2	1	8
Zhu R	2014	Beijing	2	2	2	2	2	10
Zhang XB	2014	Shanghai	2	1	2	0	2	7
Zhang QL	2014	Guangdong	2	1	2	2	1	8
Wang XH	2014	Beijing	2	0	2	2	1	7
Ji W	2013	Jiangsu	2	1	2	2	2	9
Liu CY	2013	Beijing	2	1	2	2	1	8
Feng JH	2012	Guangdong	2	2	2	2	1	9
Zhang L	2012	Beijing	2	0	2	1	1	6
Wang W	2012	Tianjin	2	0	2	2	1	7
Qian XB	2010	Zhejiang	2	2	2	2	1	9
He Y	2014	Guangdong	2	2	2	2	1	9
Liu WK	2014	Guangdong	1	2	2	2	1	8
Cai XY	2014	Guangdong	2	0	2	2	1	7
Lu Y	2013	Shandong	2	1	1	2	0	6
Huo X	2013	Hubei	1	2	2	2	2	9
Xia Q	2014	Chongqing	2	0	2	2	2	8
Zhou W	2013	Zhejiang	2	2	1	2	2	9
Zhang Q	2013	Jiangsu	2	2	1	1	2	8
Zhang XL	2013	Jiangsu	2	0	2	2	2	8
Zhang C	2013	Beijing	2	2	2	2	2	10
Huang GH	2013	Gansu	1	2	2	2	1	8
Lu Y	2013	Shandong	2	2	1	2	1	8
Qin X	2013	Chongqing	2	1	2	2	1	8

Xiang Z	2013	Beijing	1	2	2	2	2	9
Yu X	2012	Beijing	1	2	2	2	2	9
Jin Y	2012	Gansu	2	1	2	2	1	8
Wang W	2010	Shanghai	2	2	2	2	1	9
Zhang RF	2010	Gansu	1	1	2	2	2	8
Zhang ZY	2010	Chongqing	1	1	2	2	2	8

*Q1: Did the study report patients' characteristics?

Q2: Did the study report diagnosis criteria of acute respiratory infection?

Q3: Did the study report specimen collection methods?

Q4: Did the study report pathogen detection methods?

Q5: Did the study report statistical methods?
